# New method of sudomotor function measurement to detect microvascular disease and sweat gland nerve or unmyelinated C fiber dysfunction in adults with retinopathy

**DOI:** 10.1186/s40200-017-0307-5

**Published:** 2017-06-12

**Authors:** John E. Lewis, Steven E. Atlas, Ammar Rasul, Ashar Farooqi, Laura Lantigua, Oscar L. Higuera, Andrea Fiallo, Lianette Laria, Renata Picciani, Ken Wals, Zohar Yehoshua, Armando Mendez, Janet Konefal, Sharon Goldberg, Judi Woolger

**Affiliations:** 1 Department of Psychiatry & Behavioral Sciences, University of Miami Miller School of Medicine, 1120 NW 14th Street Suite #1482A, Miami, FL 33136 USA; 2 Department of Medicine, University of Miami Miller School of Medicine, Miami, FL USA; 3 Laria Eye Care, Miami, FL USA; 4 Aran Eye Associates, Miami, FL USA; 5 Department of Ophthalmology, University of Miami Miller School of Medicine, Miami, FL USA; 6 Department of Family Medicine and Community Health, University of Miami, Miami Miller School of Medicine, Miami, FL USA; 7 Glow Health PA, Bay Harbor Islands, FL USA

**Keywords:** Retinopathy, ANS-1, Sudomotor test, NO Sweat Peak, iSweat Peak, Microvascular diseases, C fiber dysfunction, Diabetes complications

## Abstract

**Background:**

Diabetes-associated microvascular complications such as retinopathy and neuropathy often lead to end-organ and tissue damage. Impaired skin microcirculation often precedes the detection of other advanced diabetic complications. The ANS-1 system contains a redesigned sympathetic skin response (ANS-1 SSR) device that measures sudomotor function, a photoplethysmography sensor, and a blood pressure device to comprehensively assess cardiac autonomic neuropathy and endothelial dysfunction. The purpose of this study was to determine the relationships between the ANS-1 SSR amplitude measured at the: (a) negative electrode (Nitric Oxide [NO] Sweat Peak) with microvascular diseases and associated vascular blood markers and (b) positive electrode (iSweat Peak) with C fiber function.

**Methods:**

All participants (healthy controls *n* = 50 and retinopathy patients *n* = 50) completed the ANS-1 system evaluation and a basic sociodemographic and medical history questionnaire, including a quality of life measure (SF-36). A small sample of blood was drawn to determine levels of homocysteine, blood urea nitrogen (BUN), C-reactive protein (CRP), and fibrinogen. Symptoms of peripheral foot neuropathy were assessed with a scale from 1 (none) to 10 (the worst). We used Spearman rank correlations, independent samples t-tests, and receiver operating characteristic curves to determine the specificity and sensitivity of the NO Sweat Peak as a potential screening marker of retinopathy.

**Results:**

The ANS-1 System Cardiometabolic Risk Score and all indicators of quality of life on the SF-36, other than Emotional Role Functioning, were significantly worse in the retinopathy patients. The sudomotor response marker NO Sweat Peak had a sensitivity of 88% and a specificity of 68% (Area Under the Curve = 0.81, *p* < 0.0001) to detect retinopathy. The NO Sweat Peak response marker inversely correlated with BUN (ρ = −0.41, *p* < 0.0001), homocysteine (ρ = −0.44, *p* < 0.0001), fibrinogen (ρ = −0.41, *p* < 0.0001), the Cardiac Autonomic Neuropathy score (ρ = −0.68, *p* < 0.0001), and the heart rate variability Total Power (ρ = −0.57, *p* < 0.0001), and it positively correlated with the Photoplethysmography Index (PTGi; ρ = 0.53 *p* < 0.0001). The ANS-1 system sudomotor response marker iSweat Peak inversely correlated with the severity of symptoms on the peripheral neuropathy scale (ρ = −0.56, *p* < 0.0001).

**Conclusion:**

The results of the study show that this new method of measuring sympathetic skin response should be useful for detecting the earliest manifestations of microvascular disease and symptoms of C fiber dysfunction.

## Background

Impaired skin microcirculation may be among the earliest manifestations of autonomic neuropathy and often precedes the detection of other diabetic complications [1]. The diabetic microvascular complications of nephropathy, retinopathy, and neuropathy collectively represent an enormous public health problem in North America. Diabetic nephropathy is the primary basis of end stage renal disease leading to hemodialysis. Diabetic retinopathy is the main origin of blindness, and neuropathy is the leading cause of lower extremity pain and amputation [2]. Microvascular complications such as peripheral neuropathy and retinopathy tend to coexist, especially in patients with diabetes [3]. For example, one study reported that 78.1% of patients with retinopathy had peripheral neuropathy [4]. Sudomotor dysfunction may be caused by cholinergic fiber neuropathy and is associated with chronic pain and/or tingling in the toes [5]. Because microvascular complications are often asymptomatic, early detection is crucial, as it allows for earlier treatment, with the goals of preventing end organ damage and limb loss and preserving quality of life (QoL).

Besides being a huge health problem, microvascular complications of diabetes are also costly. The CODE-2 study in the United Kingdom revealed that the presence of microvascular complications increased the total treatment costs of diabetes by 2.5-fold compared to patients without these problems [6]. A similar study conducted in Germany demonstrated that foot ulcer and amputation increased treatment expenditure by 4-fold and 6-fold, respectively [6].

Standard guidelines for diabetes control and care increasingly recommend early detection of complications to maximize treatment options [7]. However, the detection of microvascular complications is difficult for several reasons. First, the initial phase of microvascular disease is generally asymptomatic. Second, a comprehensive evaluation is time consuming and expensive, potentially involving collaboration among specialists in ophthalmology, neurology, nephrology, and/or endocrinology. For example, a comprehensive evaluation for peripheral neuropathy, if undertaken fully, is invasive and expensive, as it may include: electrodiagnostic testing to measure the electrical activity of muscles and nerves, such as electromyography, nerve conduction velocity and nerve biopsy, spinal tap, magnetic resonance imaging, and/or blood tests to check for vitamin deficiencies, toxic exposures, and immune dysfunction. Thus, a rapid, non-invasive, accurate test to detect early microvascular disease complications in the primary care setting is urgently needed. Such a test would likely improve patient outcomes and lower health care costs [8].

The ANS-1 system contains a sympathetic skin response (SSR) device to non-invasively screen for the small cholinergic fiber density and microcirculation state [9–11]. Previously, we compared the accuracy of the ANS-1 system versus validated, standardized assessments to detect type 2 diabetes. Our studies were conducted without any adverse events or complications in using the system [12].

## Methods

### Aim

The purpose of this study is to assess the accuracy of the ANS-1 system to detect microcirculatory disorders, inflammatory markers, and peripheral neuropathy symptoms in patients with retinopathy, since these conditions may often coexist.

### Study design and setting

The study was conducted with the approval of the University of Miami Institutional Review Board for human subjects research.

### Participants

Potential participants (*n* = 142) were identified through referrals to the University of Miami Miller School of Medicine from November 2015 to January 2017. Out of 108 eligible participants, 50 healthy subjects and 50 patients with retinopathy were enrolled in the study, and their data were used for analysis. Six subjects in the healthy group were later confirmed to have neuropathy, one subject in the healthy group had blood drawn with the wrong tube, and one retinopathy patient’s ANS-1 assessment failed. Thus, we excluded the data from these 8 participants.

### Inclusion and exclusion criteria

Inclusion criteria were: (a) 18+ years of age for patients with retinopathy and 40+ years of age for healthy participants; (b) English or Spanish speaking; (c) ability to provide written informed consent; and (d) willingness to have blood drawn. Exclusion criteria were: (a) women who were pregnant; (b) for healthy subjects, kidney or thyroid disease, peripheral neuropathy, or taking any anti-inflammatory medication current use of alpha or beta blockers or corticosteroids; or (c) contraindications to the use of the ANS-1 system, including presence of an automatic implantable defibrillator, erratic, accelerated, or mechanically-controlled irregular heart rhythms, arterial fibrillation/flutter, or any implanted electronic device.

### Description of the ANS-1 system

The ANS-1 is an FDA cleared and patented system (LD Technology, Inc., Miami, FL, USA) that contains three modules: (a) a SSR device, (b) a plethysmography sensor, and (c) a blood pressure device. The SSR device was named SudoPath, and the plethysmography sensor and blood pressure device were named TM-Oxi before the FDA clearance of the ANS-1 system. In addition, the ANS-1 system replaced the ES Complex system (LD Technology, Inc.) and integrated the ES Complex system algorithms for the photoplethysmography analysis.

The ANS-1 SSR module assesses sudomotor function by generating a low-voltage signal with a constant weak direct current (DC) that is fed to the active electrode. DC passes through the polysynaptic reflex, and upon reaching the pre- and post-ganglionic sympathetic sudomotor fibers it increases the skin electrical current in contact with the passive responsive electrode.

**Fig. 1:**
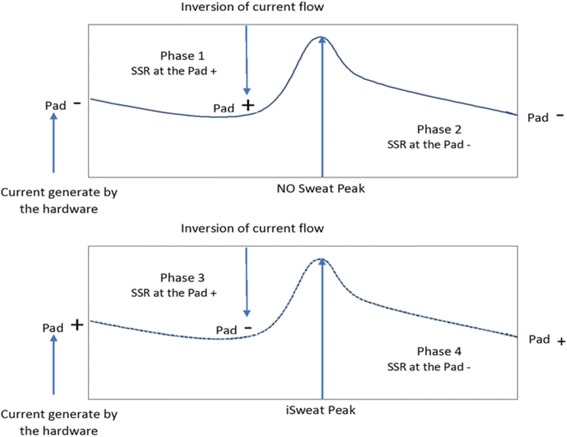
Phases of Low Voltage Signal with Constant Weak DC Applied to Electrode in the Sequence of ANS-1 SSR System. Note: Pad - = Negative electrode and Pad + = Positive electrode.

The ANS-1 SSR uses two electrode pads placed under the soles of the feet, where sweat gland density is very high. A low voltage signal with a constant weak DC current is applied to the electrode in the following sequence (see Fig. 1). *(Phase 1)* The current is sent from the negative to the positive electrode during 30 s. The voltage is positive. *(Phase 2)* The polarity of the voltage is switched from the positive to negative electrode for the next 30 s. The voltage is negative with no ion, and the peak of voltage is measured. This peak is named Nitric Oxide (NO) Sweat Peak because it reflects the effect of acetylcholine on skin blood flow. *(Phase 3)* The current is sent from the positive electrode to negative electrode. The voltage is negative. *(Phase 4)* The polarity of the voltage is switched from the negative to the positive electrode for the next 30 s. The voltage is positive with a higher ion concentration (OH-), and the peak of voltage is measured. This peak is named iSweat Peak because it reflects the second effect of acetylcholine on the sweat glands. The NO Sweat Peak normal range is ≥832 millivolts (mV). The iSweat Peak normal range is ≥1000 mV. The total time of the measurement is 2 min. A recent study found that the ANS-1 SSR module had a sensitivity of 91.4% and a specificity of 79.1% to detect peripheral distal neuropathy symptoms and was correlated (*r* = 0.68, *p* < 0.0001) with the CAN score [13].

The plethysmography sensor is used to detect heart rate (from the first derivative of the photoplethysmography [PTG] waveform) to assess cardiac sympathetic nervous system (SNS) function and parasympathetic nervous system (PNS) function. The assessment is first performed at rest by using Heart Rate Variability (HRV) analysis algorithms and subsequently assessed by performing the Cardiac Autonomic Reflex Tests (CART), Valsalva maneuver, deep breathing, and change in posture, which also involve blood pressure measurements [12, 14]. The HRV analysis provides markers of SNS and PNS regulation, such as Total Power and the Stress Index. A Cardiac Autonomic Neuropathy (CAN) score is calculated from the results of the CART. Each test is scored as 0 = normal, 1 = borderline, or 2 = abnormal. The Total Power normal range is ≥780 milliseconds squared (msec^2^). The Stress Index normal range is ≤180%. The CAN score normal range is <2, and a CAN score ≥4 is considered diagnostic of cardiac neuropathy.

In addition, ANS-1 performs a PTG spectral analysis, using Fast Fourier Transforms (FFT) of the total records of the oximeter wave form providing 3 frequencies: high (HF), low (LF), and very low frequencies (VLF). The PTG Total Power (PTG-TP) is the sum of the three surfaces or powers of VLF, LF, and HF expressed in msec^2^ units. The PTG-TP normal range is <406 msec^2^.

Previously, the ANS-1 PTG-TP was compared with insulin resistance measured by the hyperinsulinemic glycemic clamp technique. The spectral analysis marker PTG-TP had a sensitivity of 95.7% and a specificity of 84.4% for identifying insulin resistance and was correlated with insulin sensitivity (*r* = −0.64, *p* < 0.0001) [15]. The PTG index (PTGi) is the sum of the amplitude of the same three frequencies expressed in volts per second (V/s) units. The PTG VLF index (PTG VLFi) is the area of the VLF frequency divided by NO Sweat Peak expressed in msec^2^/microsiemens (msec^2^/μSi) units. The PTGi normal range is ≥40 V/s, and the PTG VLFi normal range is ≥33 msec^2^/μSi.

Finally, the ANS-1 Cardiometabolic Risk Score (CMRS) is calculated using the following variables: systolic and diastolic blood pressure, body mass index (BMI), HRV, and PTG spectral analysis indicators. Each variable is scored as 0 = normal, 1 = borderline, or 2 = abnormal to calculate the CMRS. The CMRS normal range is <4.

Our recent study showed that the ANS-1 CMRS and PTGi had significant correlation with a 2-h oral glucose tolerance test (OGTT) for glucose (*r* = 0.56, *p* < 0.001) and was significantly different between type 2 diabetes patients and healthy participants [12]. Another recent study [16] using the ANS-1 system found that the PTGi (specificity = 86.1% and sensitivity = 87.3%) and PTG VLFi (specificity = 86.1% and sensitivity = 93.6%) markers were able to detect endothelial dysfunction in coronary artery disease (CAD) patients. In practice, the ANS-1 system is focused on early detection of complications of chronic disease, such as type 2 diabetes, which affect the autonomic nervous system and vascular function at different levels.

### ANS-1 assessment

As per standard protocol, every team member was trained by the manufacturer. Participants were instructed to sit in a chair in front of the ANS-1 unit with no shoes and socks, feet on the metal plates, right index finger in the pulse oximeter, and blood pressure cuff on the left arm. Each subject relaxed for at least 5 min prior to assessment in our office with comfortable temperatures 70–74° Fahrenheit at ~70% humidity.

After inputting demographic, anthropometric, and physical activity information, the assessment was initiated for 2 min while the participant was sitting down. Then, the participant was asked to perform the Valsalva maneuver by squeezing the nose with the left hand while trying to breathe out for 15 s with the mouth closed to build pressure. After releasing the nose, the participant was told to breathe deeply for 30 s, inhaling and exhaling for 5 s each. Finally, the participant was required to stand up until the assessment was completed, while straightening the left arm to the side and keeping the index finger in the pulse oximeter. The entire assessment lasted about 12 min.

### Biomarkers

General inflammation was assessed with serum C-reactive protein (CRP), fibrinogen, and homocysteine; key indicators of multiple inflammatory processes. Kidney function was assessed with blood urea nitrogen (BUN) and creatinine levels. Venous blood (one 6 mL EDTA tube) was collected from participants by a certified phlebotomist, and serum was obtained after allowing the blood to clot for 30 min followed by centrifugation at 1500 × *g* for 15 min at 4° Celsius. The samples were sent the same day for analysis to the biochemistry laboratory. All analytes were measured on a Roche Cobas 6000 auto-analyzer (Roche Diagnostics, Indianapolis, IN, USA) using manufacturers reagents and following all manufacturer’s instructions for instrument set up and performing the assays. All tests had intra- and inter-assay coefficients of variation <5%.

### Questionnaires

Sociodemographic and health history data were collected using questionnaires that inquired about gender, marital status, race/ethnicity, education, income, current and previous medical diagnoses, injuries and hospitalizations, medication and dietary supplement use, sleep, and behaviors such as coffee, alcohol, and tobacco use. A general peripheral neuropathy questionnaire was used to assess the location, onset, provocation, quality, radiation, severity, and temporality of the condition. The SF-36v2™ Health Survey was utilized to provide psychometrically-based physical and mental health summary measures and a preference-based health utility index [17]. It is a generic measure that does not target a specific age, disease, or treatment group.

### Statistical analysis

Data were analyzed using SPSS 24 (IBM Inc., Chicago, IL, USA) for Windows. Frequency and descriptive statistics were calculated on all variables. We used Spearman rank correlations between the ANS-1 system variables and the biomarkers. We used one-way analysis of variance to differentiate healthy subjects and retinopathy patients on physical data and biomarkers. We used independent samples t-tests to compare healthy subjects to retinopathy patients on the ANS-1 CMRS variable and the scales on the SF-36. Healthy subjects and retinopathy patients were compared with receiver operating characteristic curves to determine the specificity and sensitivity of the ANS-1 response marker NO Sweat Peak as a potential screening marker of retinopathy. The criterion for statistical significance was α = 0.05.

## Results

### Adverse events

No adverse events were reported during the study with the use of the ANS-1 system or while collecting other information.

### Sociodemographic and health profile

Table 1 displays the sociodemographic and health characteristics for each study group. One hundred subjects (*n* = 50 healthy controls and *n* = 50 patients with retinopathy) were enrolled in the study with analyzable data. Of the retinopathy subjects, 3 had type 1 diabetes and 45 had type 2 diabetes. The retinopathy patients were significantly older, more likely to be Hispanic, and had higher BMI, systolic and diastolic blood pressures, and homocysteine, fibrinogen, BUN, and creatinine levels (see Table 1). Although the CRP value for the retinopathy patients was approximately 6 points higher than the healthy controls, the difference was not statistically significant. However, the CRP data were non-normal and were log-transformed. After log transformation, we found a significantly higher CRP value for the retinopathy patients compared to the healthy subjects. Table 2 displays the ANS-1 system marker CMRS and the scales on the SF-36. The CMRS was significantly lower in the healthy controls, and all SF-36 scales showed significantly better QoL, other than Emotional Role Functioning, in the healthy controls.

**Table 1:** Sociodemographic and Health Characteristics of the Sample

Variable	Category	Healthy controls (*n* = 50)	Retinopathy patients (*n* = 50)	Statistic, *p* value
Age	-	52.9 ± 10.9 (40, 80)	63.9 ± 13.9 (26, 87)	F(1, 99) = 19.3, *p* < 0.001
Gender	Male	17 (34%)	18 (36%)	χ^2^(1) = 0.04, *p* = 0.83
	Female	33 (66%)	32 (64%)	
Race/Ethnicity	White, non-Hispanic	18 (36%)	4 (8%)	χ^2^(3) = 14.4, *p* = 0.002
	Black, non-Hispanic	17 (34%)	16 (32%)	
	Hispanic	15 (30%)	29 (58%)	
	Other	0	1 (2%)	
BMI (kg/m^2^)	-	26.3 ± 4.5 (17.4, 38.4)	29.2 ± 6.2; (18.3, 45.4)	F(1, 99) = 7.3, *p* = 0.008
SBP (mm Hg)	-	125.2 ± 16.4 (83, 160)	150.6 ± 23.6 (105, 218)	F(1, 99) = 39.2, *p* < 0.001
DBP (mm Hg)	-	72.9 ± 12.4 (55, 100)	80.3 ± 14.4 (59, 144)	F(1, 99) = 7.7, *p* = 0.007
Homocysteine (μmol/L)	-	10.4 ± 3.0 (6.0, 19.6)	15.0 ± 4.6 (7.1, 26.8)	F(1, 99) = 35.0, *p* < 0.001
Fibrinogen (mg/dL)	-	461.9 ± 116.6 (263, 889)	567.2 ± 145.5 (340, 1039)	F(1, 99) = 16.0, *p* < 0.001
CRP (mg/L)	-	2.6 ± 5.5 (0.14, 35.9)	8.7 ± 28.5 (0.14, 186.7)	F (1, 99) = 9.6, *p* < 0.01
BUN (mg/dL)	-	13.9 ± 4.1 (6, 27)	19.3 ± 6.9 (8, 36)	F(1, 99) = 22.1, *p* < 0.001
Creatinine (mg/dL)	-	0.8 ± 0.21 (0.4, 1.3)	0.96 ± 0.57 (0.4, 4.5)	F(1, 99) = 3.3, *p* = 0.07

Note: Continuous data are represented by M ± SD (R)

*BMI* Body mass index, *SBP* Systolic blood pressure, *DBP* Diastolic blood pressure, *CRP* ﻿C-reactive protein﻿, *BUN* Blood urea nitrogen

**Table 2:** Cardiometabolic Risk on the ANS-1 and Quality of Life on the SF-36

Variable	Healthy controls (*n* = 50)	Retinopathy patients (*n* = 50)	Statistic, *p* value
Cardiometabolic Risk Score	4.2 ± 3.6 (0, 13)	7.7 ± 3.3 (2, 14)	t(98) = 5.2, *p* < 0.001
Physical Functioning	90.1 ± 20.6 (0, 100)	55.4 ± 30.7 (0, 100)	t(98) = 6.6, *p* < 0.001
Physical Role Functioning	89.0 ± 21.9 (0, 100)	66.1 ± 29.1 (0, 100)	t(98) = 4.4, *p* < 0.001
Emotional Role Functioning	88.8 ± 25.0 (0, 100)	81.3 ± 26.6 (0, 100)	t(98) = 1.5, *p* = 0.15
Mental Health	83.6 ± 15.1 (30, 100)	75.2 ± 22.5 (10, 100)	t(98) = 2.2, *p* = 0.03
Social Functioning	89.3 ± 16.4 (50, 100)	75.0 ± 30.4 (0, 100)	t(98) = 2.9, *p* < 0.01
Vitality	76.3 ± 18.7 (12.5, 100)	64.1 ± 23.0 (18.75, 100)	t(98) = 2.9, *p* < 0.01
Bodily Pain	80.1 ± 16.8 (22, 90)	57.6 ± 27.9 (0, 90)	t(98) = 4.9, *p* < 0.001
General Health	84.9 ± 19.1 (20, 100)	57.1 ± 25.6 (5, 92)	t(98) = 6.2, *p* < 0.001

Note: Continuous data are represented by M ± SD (R)

### Correlations with NO Sweat Peak and other markers

The ANS-1 response marker NO Sweat Peak inversely correlated with BUN (ρ = −0.41, *p* < 0.0001), homocysteine (ρ = −0.44, *p* < 0.0001), fibrinogen (ρ = −0.41, *p* < 0.0001), the CAN score (ρ = −0.68, *p* < 0.0001), and HRV Total Power (ρ = −0.57, *p* < 0.0001), and it positively correlated with PTGi (ρ = 0.53 *p* < 0.0001).

### Receiver operating characteristic curve of NO Sweat Peak

**Fig. 2:**
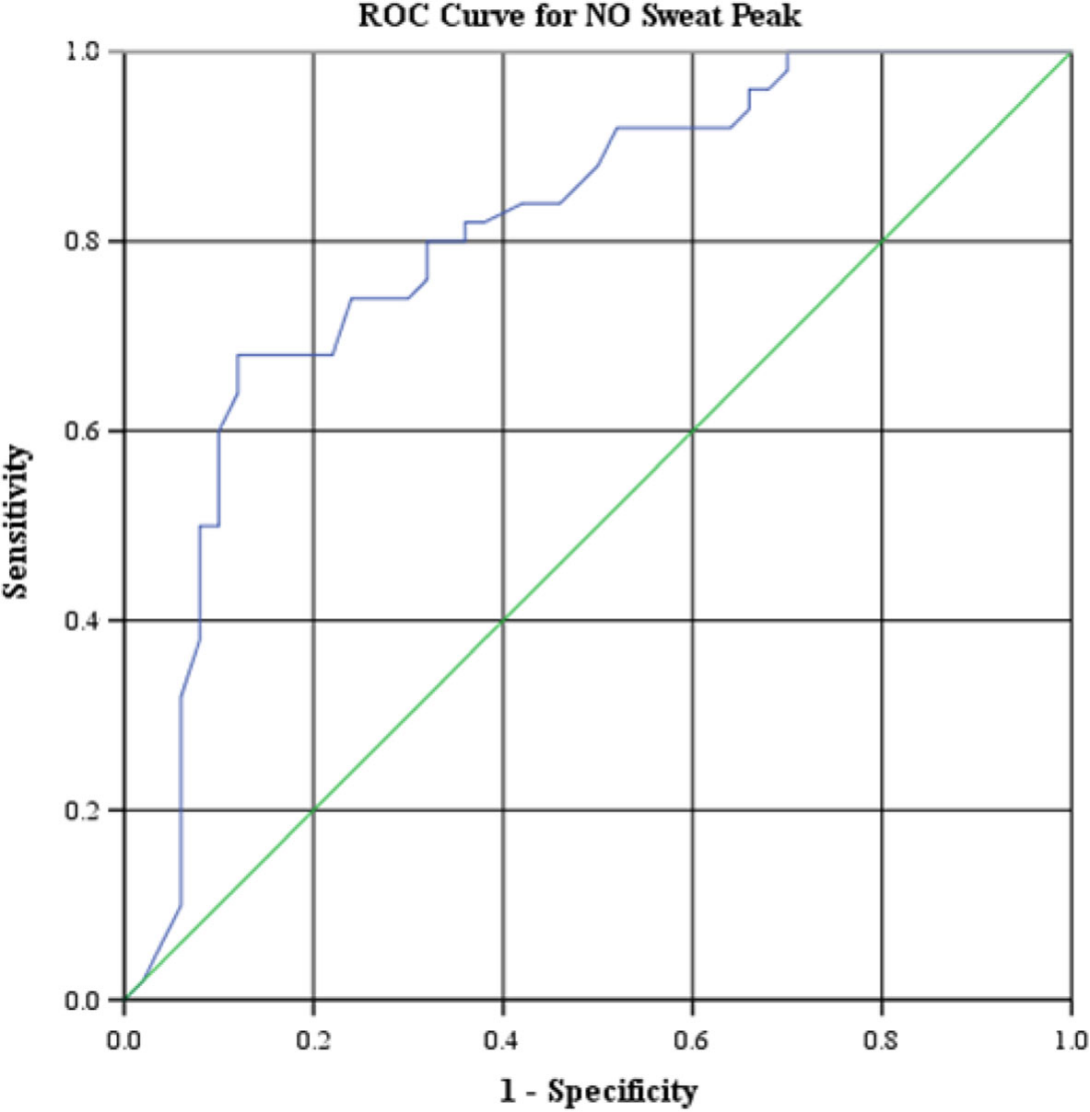
Receiver Operating Characteristic Curve (Sensitivity and Specificity) for the Nitric Oxide Sweat Peak

NO Sweat Peak had a sensitivity of 88% and specificity of 68% (cut-off score ≤ 64) with an area under the curve (AUC) = 0.81 (SE = 0.04; 95% CI = 0.72, 0.88) and an asymptotic significance <0.0001 (see Fig. 2).

### Correlations among other markers

The ANS-1 system sudomotor response marker Sweat Peak inversely correlated with the severity of symptoms on the peripheral neuropathy scale (ρ = −0.56, *p* < 0.0001). The ANS-1 system marker Stress Index and the CAN score correlated with CRP (ρ = −0.38, *p* < 0.0001 and ρ = 0.40, *p* < 0.0001, respectively), iSweat Peak correlated with PTG-TP (ρ = 0.48, *p* < 0.0001), and PTG VLFi correlated with fibrinogen (ρ = 0.43, *p* < 0.0001).

## Discussion

Traditional and recognized neurophysiologic measurements of sudomotor function include thermoregulatory sweat testing, quantitative sudomotor axon reflex testing, silicone impressions, and SSR. Sudomotor testing is widely used to assess the integrity of peripheral and central autonomic functions. In addition, clinical data suggest that sudomotor testing may be the most sensitive means to detect peripheral small fiber neuropathy [9].

In the present study, NO Sweat Peak was correlated with: (a) retinopathy, a microvascular disease, (b) BUN, an indicator of kidney function (diabetic nephropathy is a microvascular disease), (c) homocysteine, which indicates a deficiency of vitamin B_12_ and microcirculation deficiencies, (d) fibrinogen, an indicator of blood clotting, (e) PTGi, a marker of endothelial function, (f) the CAN score, and (g) HRV Total Power, which are markers of autonomic neuropathy. All of these significant findings strongly indicate that NO Sweat Peak is a marker of microvascular disease. Sweat Peak was correlated with the severity of symptoms of peripheral neuropathy. This finding suggests that iSweat Peak is a marker of sweat gland nerve or unmyelinated C fiber dysfunction or damage. Thus, the results of the current study help to support the validity of our assumptions regarding the ANS-1 response markers NO Sweat Peak and iSweat Peak.

A paucity of information has been published in the extant literature with other methods of evaluating sudomotor function. Comparing the NO Sweat Peak ability to discriminate microvascular disorders using a sudomotor test, only one other study showed that a galvanic skin response measurement had a lower AUC of 0.65 and 0.68 for the feet and hands, respectively, than NO Sweat Peak (AUC of 0.81) [18]. Comparing the Sweat Peak ability to detect the severity of symptoms of peripheral neuropathy using a sudomotor test, the Neuropad test was more highly correlated (ρ = 0.66) with symptoms of peripheral neuropathy, but not on the severity of the symptoms [19]. Moreover, to our knowledge the ANS-1 system has been more widely studied in comparison to more biomarkers than other devices in the marketplace.

The underlying method used by the ANS-1 SSR is based on sudomotor function background and physics. Sudomotor function is primarily mediated by the stimulation of the post-sympathetic cholinergic fibers through the action of acetylcholine. Acetylcholine stimulates the nicotinic muscarinic (M) receptors. The activation of M receptors has 2 effects: (a) Activation of M3 on vascular endothelial cells causes increased synthesis of NO, which diffuses to adjacent vascular smooth muscle cells and causes their relaxation and vasodilation of the vessels that allows an exchange between vessels and sweat glands and facilitates the production of sweat [1]. (b) Activation of inositol polyphosphates (IPP) causes intracellular calcium mobilization and calcium influx and ions diffusion into the lumen of the sweat duct, which affects the sweat nerve glands (unmyelinated C fiber) function or density [20].

Therefore, sudomotor dysfunction, defined as decreased sudomotor activity, could be reflective of impaired C fiber function or density (low level or absence of acetylcholine production) or impaired skin microcirculatory vasodilation (low or absence of response to NO) due to endothelial dysfunction. When a constant DC is applied to the human body between two electrodes, the current is carried by ions, ions flow from the negative electrode toward the positive electrode, and the current flow is opposite of the flow of ions. Therefore, the positive polarity corresponds to a higher concentration of ions [21].

Since sweat is 96.4% water, the concentration of hydroxide ions (OH-) at the positive electrode is the same (99.6%) when the current is applied. Activation of IPP causes a calcium influx and movement of negative ions across the apical membrane. However, since OH- and chloride (Cl-) are in competition, the very low concentration of Cl- is not significant and does not affect the measurement, and only the OH- concentration reflects the IPP activation [22]. Therefore, the measuring response at the positive electrode (iSweat Peak) is related to the effect of acetylcholine on sweat nerve glands (unmyelinated C fiber) function or density, and the response at the positive electrode is related to the effect of acetylcholine by axon reflex mediated vasodilation on skin microcirculation.

The correlations of the HRV marker Stress Index and CAN score with CRP suggest that autonomic nervous system dysfunction is an inflammatory process, which has been shown previously, as baroreflex sensitivity was inversely correlated with both white blood cell count (WBCC) and CRP, and HRV was independently inversely associated with WBCC [23]. As inflammation has been widely established as increasing cardiovascular disease risk [24–26], our finding at least partially explains the pathway of autonomic neuropathy for increasing such risk. More research needs to be performed to validate our result.

Our discovery that the correlation between the SSR marker Sweat Peak with the PTG-TP marker of insulin resistance suggests that the sweat gland nerve or unmyelinated C fiber function is influenced by insulin production. To our knowledge, the ANS-1 SSR module is the only sudomotor test that can distinguish between microcirculatory and autonomic nervous system disorders in one assessment. Sudomotor dysfunction or decreased activity could be reflective of impaired C fiber function or density (low level or absence of acetylcholine production) or impaired skin microcirculatory vasodilation (low or absence of response to NO) due to endothelial dysfunction [21]. Other sudomotor tests require the inclusion of laser-Doppler flowmetry. Several studies using laser-Doppler flowmetry demonstrate the benefit of assessing skin microcirculation for risk of diabetes or for diabetic foot care [27–29]. Nonetheless, with the ANS-1 SSR the clinician/patient dyad does not have to utilize a more extensive and time-consuming procedure to determine sudomotor functioning, as with laser-Doppler flowmetry. Additionally, the ANS-1 SSR module demonstrates performance capabilities that should quickly and dramatically improve the detection of the earliest manifestations of microvascular and sweat gland nerve or unmyelinated C fiber dysfunction, which often precede other diabetic complications.

Although the early manifestation of microvascular diseases is asymptomatic, eventual sweat gland nerve dysfunction or damage causes chronic pain such as cold, tingling, or burning or a pins and needles sensation [30]. Diabetes and prediabetes are frequently associated with sweat gland nerve dysfunction and microvascular dysfunction; however, concomitant large fiber involvement is seen more often. Pain in sweat gland nerve dysfunction has been linked to abnormal glucose metabolism. Individuals with diabetes and metabolic syndrome appear to have twice the risk of developing sweat gland nerve dysfunction compared to those with diabetes alone [31]. Thus, the standard of care to coordinate the detection of microvascular diseases among specialists would be time consuming and expensive. Alternatively, the ANS-1 SSR would be a fast, non-invasive test at nominal cost to detect early microvascular disease complications that can be used in the primary care setting prior to referring patients to specialists in the case of abnormal results.

### Limitations

The retinopathy patient group had a greater percentage of Hispanics relative to the healthy control group. We did not correlate the severity of retinopathy (mild, moderate, or severe non-proliferative or proliferative types) with the ANS system markers. We did not measure vitamin B_12_ or urine microalbumin to compare to the ANS system markers. The ANS-1 system may be useful in guiding screening for retinopathy and other microvascular dysfunction prior to overt diabetes or cardiovascular disease, but larger scale studies are warranted to substantiate these results and to continue to explore the clinical and prognostic significance of the system.

## Conclusion

The results of the current study showed that the ANS-1 process of measuring SSR by using electrical stimulation with a change of DC flow direction during the exam and showing the peak amplitudes of current at the positive and negative electrodes should be useful for detecting the earliest manifestations of microvascular diseases and sweat gland nerve or unmyelinated C fiber dysfunction. Our study also found that NO Sweat Peak had adequate sensitivity and specificity to distinguish between healthy controls and patients with retinopathy and additionally showed a lower QoL and higher CMRS among the retinopathy patients. NO Sweat Peak was also inversely correlated with: (a) BUN, which estimates kidney functioning, (b) homocysteine, which is an inflammatory marker that is indicative of vitamin B_12_ or folic acid deficiency, and (c) the CAN score. We also found elevated CRP and fibrinogen levels in the retinopathy group, suggesting increased inflammation and cardiovascular disease risk. Given the correlations between the HRV marker Stress Index and CAN score with CRP, the ANS-1 system may also prove to be an excellent screening tool for early cardiovascular disease and/or an assessment of cardiovascular risk.

Since retinopathy and kidney disorders are microvascular diseases, and supplementing with vitamin B_12_ is known to improve microcirculation, NO Sweat Peak could be useful in identifying microcirculatory disorders. Thus, it should be considered a microcirculation marker.

Moreover, since NO Sweat Peak, a marker of sudomotor function and microcirculatory disorders, is highly correlated with the CAN score, a marker of autonomic neuropathy, the assumption that a microcirculatory disorder is related to autonomic neuropathy seems to be supported by the results of our study. This finding suggests a new way to prevent and treat autonomic neuropathy by early detection with the ANS-1 system and treatment of microcirculatory disorders with vitamin B_12_, folic acid, or other anti-inflammatory nutrients or foods. Finally, based on the composite findings of several studies using the ANS-1 markers in 3 continents by detecting (a) insulin resistance and correlating with OGTT and (b) microcirculatory disorders, symptoms of peripheral neuropathy, cardiac autonomic neuropathy, and CAD [12–16], the ANS-1 system has great value in screening for pre-diabetes and in the early detection of diabetes, when treatment options are available and may delay or reverse the disease and its complications.

## Abbreviations

ANS-1 SSR: ANS-1 sympathetic skin response
AUC: Area under the curve
BMI: Body mass index
BUN: Blood urea nitrogen
CAD: Coronary artery disease
CAN: Cardiac autonomic neuropathy
CART: Cardiac autonomic reflex test
Cl-: Chloride ion
CMRS: Cardiometabolic risk score
CRP: C-reactive protein
DC: Direct current
ES: Electro-sensor
ESC: Electro-sensor complex
FFT: Fast Fourier transform
HF: High frequency
HRV: Heart rate variability
IPP: Inositol polyphosphate
LF: Low frequency
M receptors: Nicotinic muscarinic receptors
NO: Nitric oxide
OGTT: Oral glucose tolerance test
OH-: Hydroxide ions
PNS: Parasympathetic nervous system
PTG: Photoplethysmography
PTGi: Photoplethysmography index
PTG-TP: PTG total power
QoL: Quality of life
SNS: Sympathetic nervous system
SSR: Sympathetic skin response
VLF: Very low frequency
WBCC: White blood cell count
